# Assessing embodied interpersonal emotion regulation in somatic symptom disorders: a case study

**DOI:** 10.3389/fpsyg.2015.00068

**Published:** 2015-02-10

**Authors:** Zeynep Okur Güney, Heribert Sattel, Daniela Cardone, Arcangelo Merla

**Affiliations:** ^1^Department of Psychosomatic Medicine and Psychotherapy, Klinikum rechts der Isar, Technical University of Munich, MunichGermany; ^2^Department of Psychology, University of Kassel, KasselGermany; ^3^Institute of Advanced Biomedical Technologies, G. d’Annunzio University Foundation, ChietiItaly; ^4^Department of Neuroscience, Imaging and Clinical Sciences, G. d’Annunzio University, Chieti-PescaraItaly

**Keywords:** emotion regulation, somatic symptom disorders, interpersonal interactions, embodiment, anger, relaxation

## Abstract

The aim of the present study was to examine the intra- and interpersonal emotion regulation of patients with somatic symptom disorders (SSDs) during interactions with significant others (i.e., romantic partners). We presented two case couples for analysis. The first couple consisted of a patient with SSD and his healthy partner, whereas the second couple consisted of two healthy partners. The couples underwent an interpersonal experiment that involved baseline, anger and relaxation tasks. During each task, partners’ cutaneous facial temperature, heart rate and skin conductance levels were measured simultaneously. Participants’ trait-emotion regulation, state-affect reports for self and other, and attachment styles were also examined. The experimental phases were successful in creating variations in physiological processes and affective experience. As expected, emotion regulation difficulties predicted higher increase in the course of temperature at each phase. Besides, the patient showed restricted awareness and reflection to emotions despite his higher autonomic activity compared to healthy controls. Both partners of the first couple revealed limited ability in understanding the other’s emotions, whereas the second couple performed relatively better in that domain. The temperature variations between the patient and his partner were significantly correlated while the correlations of temperature changes between the second couple were negligible except anger task. The study supported the merits of an embodied interpersonal approach in clinical studies. The tentative results of the cases were discussed in the light of findings in emotion regulation and attachment research.

## INTRODUCTION

Somatic symptom disorder (SSD) is characterized by persistent somatic disturbances, which cause severe impairment in patients’ daily life (DSM-V). The disturbances are accompanied by excessive and dysfunctional thoughts, affects, behaviors or health concerns. Psychological factors contribute to the development, course and treatment of these disorders (Henningsen et al., 2003; Sattel et al., 2012). The overlap of multiple somatic symptoms, comorbidity with psychiatric and psycho-social disturbances, absence of clear diagnoses and ineffective treatments make SSD both difficult to treat and costly for society (Wessely et al., 1999; Henningsen et al., 2007). Such an overlap of multiple physical and psychological symptoms renders SSD as being neither purely physical nor mental but truly psychosomatic (Wessely et al., 1999; Henningsen et al., 2007).

An increasing number of studies highlight the presence of emotion regulation disturbances in SSD, such as emotion suppression (Burns et al., 2011; Gul and Ahmad, 2014), rumination, catastrophizing (Hadjistavropoulos and Craig, 1994; Garland et al., 2011), decreased ability to up-regulate positive emotions (Zautra et al., 2001), imbalance in physiological arousal (Pollatos et al., 2011a,b), and diminished ability in emotional awareness (Waller and Scheidt, 2004; Subic-Wrana et al., 2010) and emotion recognition (Beck et al., 2013). In addition, difficult transference and counter-transference in psychotherapy related to patients’ resistance to experience emotions was reported (Yasky et al., 2013).

### COHERENCE BETWEEN EMOTION RESPONSE SYSTEMS IN SSD

Theories that explain the nature and development of SSD put an emphasis on the role of emotion regulation disturbances (see Waller and Scheidt, 2006 for a thorough review of the theoretical models). For example, early psychodynamic theories depicted somatic symptoms as defenses of the unconscious unresolved affective conflicts (Freud, 1961). Alexander (1950), “one of the founders of psychosomatic medicine,” posited that, if affect-related physiological arousal is not realized into action, in time, it is experienced as disturbing physiological states. Deficits in symbolic affect representation, such as limited emotional awareness and ability to reflect on and describe emotions (i.e., alexithymia) were identified as typical to SSD (Sifneos, 1973; De Gucht and Heiser, 2003; Subic-Wrana et al., 2010). Similarly, an impaired integration of symbolic (language, imagery) and subsymbolic emotion schemas (sensory, somatic, and motoric forms) was asserted to feature SSD (Bucci, 1997). Attachment theories also point to the disequilibrium among stress regulating networks associated with insecure attachment style (Luyten et al., 2012). It is posited that, having internalized certain dysfunctional attachment patterns and regulation strategies, patients with SSD tend to regulate stress by employing these strategies later in life. This may lead to imbalance between stress response networks, which are associated with impairments in patients’ ability of embodied mentalization (i.e., understanding one’s own and others’ feelings and intentions, and linking these internal processes with the body; Luyten et al., 2012).

The theoretical models mentioned above as well as existing empirical research imply a pattern of emotion regulation in SSD, which is characterized by incoherence between emotion constituents. Supporting the postulation of incoherent emotional processing, a systematic review on emotion regulation in SSD (Okur et al., in revision) revealed that patients with SSD tend to detach from the emotion by means of disengaging the cognitive- behavioral components of emotion from the emotional perturbations. For instance, patients were shown to have higher levels of alexithymia and reduced ability in emotion recognition and affective theory of mind (Subic-Wrana et al., 2010; Beck et al., 2013; Castelli et al., 2013; Haas et al., 2013; Stonington et al., 2013). On the other hand, the few available studies having examined somatic components of emotions demonstrated aberrant or vigilant somatic reactivity, such as greater startle responses, paraspinal muscle reactivity, sympathetic activity or stress sensitivity in SSD (Seignourel et al., 2007; Burns et al., 2008; Twiss et al., 2009; Luyten et al., 2011; Pollatos et al., 2011a,b).

Emotion theories generally agree that emotion response system has multiple components coordinating with each other (Hollenstein and Lanteigne, 2014). The concordance among these physiological, behavioral and experiential response systems, which facilitates adaptive and coordinated responses as the emotion unfolds over time, is described as emotional coherence (Mauss et al., 2005). Although almost all emotion theories agree on some degree of coherence between the emotion response systems, empirical studies show quite mixed findings (Mauss et al., 2005; Hollenstein and Lanteigne, 2014). Recently, several theoretical and methodological issues related to emotional coherence were particularly addressed in a special issue (Hollenstein and Lanteigne, 2014). It was argued that, the inconsistent findings might be related to methodological errors such as non-correspondent timing or obstruction of concordance with individual differences, such as emotion regulation (Mauss et al., 2005; Butler et al., 2014; Hollenstein and Lanteigne, 2014). When taking precautions regarding these errors, the authors could show moderate to high coherence.

We also argue that, since emotional process is a continuous, inseparable regulating and regulated system (Davidson, 1998; Kappas, 2011), a person’s own emotion regulation patterns constantly influence the emotional coherence. Therefore, it is probable that level of coherence would vary between people having distinct patterns of emotion regulation, as would be the case in patients with SSD. In fact, some studies exist supporting the effect of emotion regulation on coherence. For example, a study comparing participants with different body awareness levels showed that experienced Vipassana meditators (awareness of visceral sensations) had the highest coherence between physiological changes and subjective experience. This was followed by experienced dancers (awareness of somatic sensations) and then controls with no experience of bodily exercises (Sze et al., 2010). Deliberately employed emotion regulation strategies affect the coherence as well. Emotion suppression was found to decrease the coherence between physiological, behavioral and experiential subsystems although acceptance of emotions was not (Dan-Glauser and Gross, 2013). Lending support to these findings, reappraisal was reported to increase the concordance for positive emotions, but to decrease it for the negative ones (Butler et al., 2014).

These findings illustrate the potential effects of emotion regulation on concordance between emotion response systems. Emotion regulation patterns, which patients with SSD unconsciously or deliberately deploy, might affect the coherence between emotional constituents. Hence, in the light of the literature on emotion regulation in SSD, we hypothesize that incoherence in emotional process characterizes the regulation patterns of patients with SSD, which is moderated by attachment and trait emotion regulation styles. This incoherent process is described by disengagement of cognitive components from the emotional perturbations but greater physiological stress responses marked by higher activity or vigilance at the somatic components of emotion. Our proposed assessment of intrapersonal emotional incoherence relies on the extent of discrepancy between emotional responses, which is manifested by restricted expression of and reflection on emotions. Simultaneously, we expect an aberrant and reactive sympathetic nervous system response.

### INTERPERSONAL REGULATION OF EMOTIONS IN SSD

Interpersonal factors, which are proposed to play a role in the development of emotion regulation disturbances in SSD, continue to trigger and maintain the psychosomatic symptoms later in life. There is quite a consensus on the role of interpersonal interactions, attachment and trauma history in dysregulated affect of SSD that is linked to alterations in the endocrine, immune, and pain regulating systems (Henningsen, 2003; Luyten et al., 2013). Lending support to this linkage, a shared neural system for social pain, such as rejection, exclusion or loss, and physical pain is acknowledged (Kross et al., 2011; Eisenberger, 2012; Landa et al., 2012).

In the developmental history of SSD, an “emotional avoidance culture” with significant adults was described, which was associated with patients’ disconnection of awareness from stress reactions in the body (Bondo-Lind et al., 2014). Besides, insecure attachment history and related impairments in interpersonal emotion regulation between the caregiver and child, such as non-expression of emotions, is commonly reported in SSD (Waller and Scheidt, 2006). Patients with SSD were reported to regulate stress by deactivating or hyperactivating attachment strategies later in life that have adverse metabolic and interpersonal consequences (Luyten et al., 2012). For example, denial of attachment needs (Luyten et al., 2012), minimization of affective experience or expression (Waller and Scheidt, 2004) or impaired embodied mentalization (Luyten et al., 2012) as well as over expression of negative affect with respect to bodily complaints and clinging behavior (Waller and Scheidt, 2006) can govern the interpersonal interactions of the patients. These dysfunctional strategies in turn generate a vicious cycle of further interpersonal distress, exacerbation of the symptoms, and further stress and symptoms (Luyten et al., 2012). Such regulation strategies can be linked to incoherence among emotion response systems. For example, in subjects with high avoidant attachment, discordance between psychological and endocrine stress measures was found. However, in subjects with low avoidant attachment, these measures were significantly correlated (Ditzen et al., 2008).

Although studies exist having examined the perceived social interactions with significant others in SSD, there is a scarce literature on how on going affects during patients’ interaction with significant others are co-regulated. Self-report studies show less supportive and cohesive family environment, conflicts in marital relationship (Mullins and Olson, 1990), frustration and helplessness of physicians, and rejecting behavior from significant others (Stuart and Noyes, 1999). A few available studies focusing on the dynamic interaction between couples have shown that interpersonal emotion regulation, such as validation or invalidation of a partner’s affective experience has predictive roles on experience of pain (Cano et al., 2008; Leong et al., 2011). A psychotherapy study has also demonstrated how affective experience of both patients and therapists influence each other, ensuing with an increased expression of negative affect (Merten and Brunnhuber, 2004). To our knowledge, no previous study has examined the dynamic coordination of physiological, experiential, and behavioral emotion response systems of patients with SSD and their interaction partners. In this study, we aim to fill in this gap by examining the relationship of affective experience, autonomic activity and trait emotion regulation of both interacting partners. Such a paradigm would facilitate a meeting of psychosomatic research with an embodied, dynamic and interpersonal approach.

We believe that studies from social cognition and developmental research on intersubjectivity can provide much insight to clinical research by introducing the constitutive aspects of social interaction, such as coordination or reciprocity. In fact, it is highlighted that the process of social interaction cannot be sufficiently grasped by examining the mere static interaction of individual elements, since social interactions possess dynamic features such as self-organization and autonomy (Di Paolo and De Jaegher, 2012). In line with such developments in social cognition, emotion regulation research has incorporated the dynamic parameters of interpersonal interactions such as emotion contagion, reciprocity, coupling, synchronicity or co-regulation, which describe the temporal emotional exchange and covariation between persons (Butler, 2011). These aspects can also uncover implicit emotion regulation patterns, which are described as processes operating free of conscious supervision (Koole and Rothermund, 2011).

In the context of these recent developments in social cognition and emotion research, we inquire how affect dysregulation takes place in interactions of patients with SSD with significant others (i.e., romantic/life partner). We propose that: (1) Intrapersonal emotional incoherence in SSD is more likely to be reciprocated by an emotional incoherence in the interaction partner. This may leave the affective exchange dysregulated and generate a system of incoherent interpersonal emotional processing. This persisting dysregulated affect at intra- and interpersonal levels might exacerbate bodily disturbances. Here, we define interpersonal emotional coherence as the correlation between interaction partners’ physiological and subjective affective response systems. (2) The parameters of autonomic nervous system activity will be less concordant during emotional interactions between dyads with SSD as compared to healthy control dyads. This concordance will be moderated by the attachment and trait emotion regulation styles of the partners.

We deemed it necessary to employ a paradigm involving real-time dyadic emotional interaction tasks (i.e., dyadic stress interview paradigms) that allows for the measurement of temporal affective exchange between persons. A base-line interpersonal task without an emotional manipulation would enable the comparison between different affective states as well as the acclimation of the participants to the experiment. Following that, an emotional interaction task that elicits a high level of arousal and negative valence, ensued by a relaxation task low in arousal and positive in valence, would permit us to examine the down- and up-regulation of emotions.

Concerning participants, comparing patient-healthy partner dyads with both healthy partner dyads would be elucidative in understanding the affective interaction patterns, that may exacerbate the symptoms, such as the reciprocal nature of dysregulated affect. In order to provide homogeneity in the sample of forthcoming studies, we aimed to focus on a certain group of SSD; somatoform pain disorder.

Anger was reported as both a particular predictor and outcome of chronic pain (Fernandez and Turk, 1995; Burns et al., 2008; van Middendorp et al., 2010). Patients’ appraisals with regard to chronic experience of pain, together with persistent treatment failures as well as not being heard by significant others and health professionals generate habitual anger in patients (Fernandez and Turk, 1995). Furthermore, high trait anger experience, as well as suppressing anger was shown to exacerbate pain through activating endocrine and muscular systems of the body (Bruehl et al., 2007; Burns et al., 2011). Therefore, anger was chosen as a central theme of the dyadic interaction task. In addition, dysfunctional regulation of positive affect was reported to be a distinctive feature of somatoform pain as opposed to “medically explained” pain (Zautra et al., 2001, 2005). In line with these findings, we aimed to examine the interplay of both down regulation of anger and up-regulation of positive affect in somatoform pain patients during interpersonal interactions. In order to activate attachment styles that would arouse characteristic emotion regulation patterns, the interaction partner was thought to be a significant other for the patient (i.e., romantic partner). In order to measure emotional coherence and affective exchange, assessment of multiple components of emotion, namely, state and trait subjective reports for emotion regulation, as well as autonomic nervous system measures were included. Below, we demonstrate the two case studies that we conducted employing our proposed paradigm.

## MATERIALS AND METHOD

### PARTICIPANTS

This study was approved by the Ethics Commission for the Faculty of Medicine of the Technical University of Munich (TUM). The first couple invited to participate in the study consisted of one patient and her partner. The patient was admitted to the Department of Psychosomatic Medicine at TUM and fulfilled the diagnostic criteria of persistent somatoform pain disorder (ICD-10 F45.40). As a comparison case, a healthy control couple who were found through the internal communication network of TUM was also recruited to the study. The first couple was between 40 and 50 years old and the second was between 30 and 40 years old.

### PROCEDURE

**FIGURE 1:**
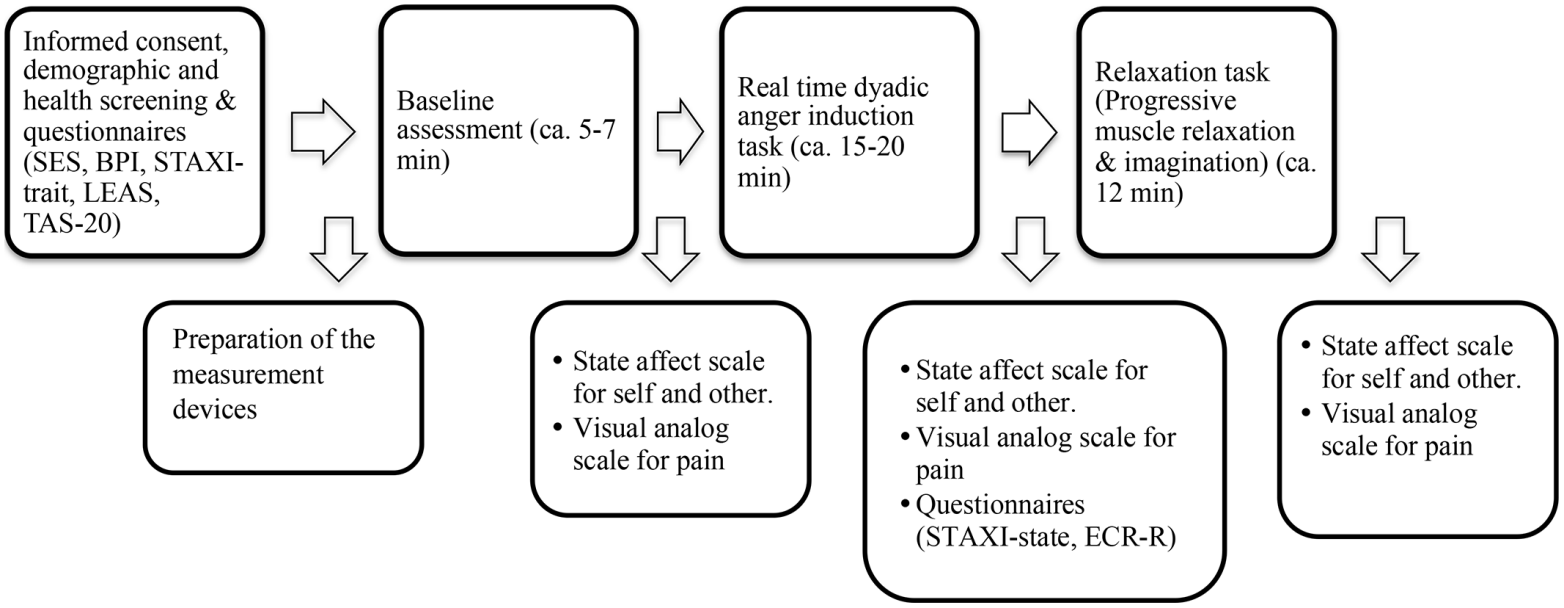
**A schematic plan of the study process.** SES, Pain Experience Scale; BPI, Brief Pain Inventory; STAXI, Spielberger Anger Expression Inventory; LEAS, Level of Emotional Awareness Scale; TAS, Toronto Alexithymia Scale; ECR, Experiences in Close Relationship-Revised.

The experiment appointments were arranged by telephone interviews. In the telephone interview, participants were screened for the existence of any medical or psychological disturbance, as well as for use of painkillers or any other medication, particularly for control purposes. Participants were asked not to take any stimulants (e.g., coffee, tea, nicotine) less than 2 h prior to testing. Upon arrival at the laboratory, couples were given oral and written briefing about the experiment and informed consent was obtained. All participants were screened for medical and psychological health status, use of medication, pain or any received treatment with an anamnestic questionnaire. The control couple and the partner of the patient did not report any health-related disturbances. Following the demographic and health screening, both partners filled in questionnaires on emotion regulation and pain experience. Thereafter, participants were invited to the experiment room and prepared for the physiological measurement. Couples underwent three phases during the experiment, which were composed of interactions with their partners. A trained interviewer, who was blind to the study hypotheses, led the couple interactions. During the entire three phases of the experiment, video recordings and physiological responses were taken of two partners. Immediately after each phase, participants reported their emotional experience and their perceptions of their partner’s emotional experience. In addition, after the dyadic anger induction task, participants were given questionnaires to assess attachment styles and state-anger experience (See **Figure 1**, for a schematic plan of the study process).

#### Emotion induction tasks

***Baseline.*** For the baseline assessment, the interviewer facilitated a 5–7 min dialog between couples about an emotionally neutral event, such as trip to the lab, events of the day or the weather as suggested by previous studies (Gottman and Levenson, 1999).

***Real-time dyadic interaction phase for anger induction.*** Compared to other methods such as movie clips or punishment tasks, interview methods have been shown to be more effective in eliciting emotions and creating physiological variations (Lobbestael et al., 2008). Furthermore, in comparison with other methods, such as showing participants pictures or videos, autobiographical recall and reliving past experiences are more effective in eliciting emotions, particularly because they are self-relevant (Ellsworth and Scherer, 2003; Kross et al., 2009). Therefore, the interview method was utilized to elicit a dynamic emergence of anger in the couples. For this task, the couples were instructed to identify a mutual past event that generated a strong feeling of anger, which could be well recalled for the experiment. One of the partners was instructed to recall and verbally describe the event. Then, both partners were invited to talk about the event, the nature of the stressor, and their thought and feelings as genuinely as possible (see Dimsdale et al., 1988). Both couples chose a conflictual topic between them, which opened up further discussions during the conversation. The interviews lasted between 15 and 20 min.

***Relaxation phase.*** After the anger induction task, participants were instructed to extricate themselves from the negative state by pursuing an audio progressive-muscle relaxation and imagination exercise that lasted ∼12 min.

### MEASURES

#### Physiological recordings

Continuous thermal imaging recordings of the face, and measures of heart rate (HR) and electrodermal activity were taken from each partner simultaneously during the entire phases of baseline, anger, and relaxation phases.

***Thermal imaging.*** Thermal imaging is a contact free method used for measuring autonomic activity manifested by variations in the cutaneous temperature, through recording of thermal infrared signals. This method was proven to be a non-invasive and robust way for measuring autonomic activity during emotional interactions (Ebisch et al., 2012; Ioannou et al., 2014). Thermal imaging was performed using two digital cameras: FLIR, SC660 (640 × 480 bolometer, FPA, sensitivity: <30 mK @ 30°C), and FLIR SC655 (640 × 480 bolometer, FPA, sensitivity: <50 mK @ 30°C). The cameras were positioned behind and just over the head of each partner, so that each camera could record the partner opposite to its position. The sampling rate was five frames/second. Variations in cutaneous temperature of the facial regions of interest were analyzed using customized Matlab programs (http://www.mathworks.com). Our primary regions of interest, the nose and the forehead were selected based on previous studies in primates and humans (Merla and Romani, 2007). After the thermal imprints were inspected visually for the recording quality, the thermograms were corrected for movement artifacts.

***Heart rate.*** Heart rate was assessed with a continuous electrocardiogram recorded with Nexus-10 equipment (Biotrace, Mind Media BV). Signals were recorded (sampled at 256 Hz) and analyzed by the computer-based Biotrace software system. A three electrodes array for each partner, which simultaneously recorded the HR of both, was used. One electrode was placed on the left and another on the right shoulder of the participant. The third electrode was placed on the left side, below the lead on the left shoulder, under the 10th rib. Before placing the electrode, the skin was cleaned to improve the quality of the signal. After the signal stabilization was achieved, data acquisition was registered. Following the collection of the data, the ECG data curves were then visually inspected for possible movement artifacts and no abnormalities were detected in any participant.

***Skin conductance level (SCL).*** Skin conductance level was recorded using the Nexus-10 device of Biotrace system, following the standard published guidelines (Boucsein, 2012). Velcro straps were attached to the II and III fingers of the participants’ non-dominant hand. Before placing the electrode, the skin was scrubbed to improve the quality of the signal. After the signal stabilization was achieved, data acquisition was registered at 32 Hz sample rate.

#### Subjective reports

Before the experiment, participants’ trait emotion regulation patterns were assessed by subjective measures of emotional awareness (Level of Emotional Awareness Scale, LEAS; Subic-Wrana et al., 2001), alexithymia (Toronto Alexithymia Scale-20, TAS-20; Bach et al., 1996) and anger regulation (Spielberger State-Trait Anger Expression Inventory, STAXI; Schwenkmezger et al., 1992). TAS is the most commonly used self-report measure of alexithymia differentiating three areas of emotion regulation difficulties: difficulty in identifying feelings, difficulty in describing feelings, and externally oriented thinking. Despite being the best-validated instrument for alexithymia, its use may be biased due to its paradoxical reliance on patients’ insight on their own ability of emotional self-reflection (Waller and Scheidt, 2004). On the other hand, LEAS is a performance-based instrument, consisting of twenty emotion-eliciting scenarios where the subjects report how they and the other person in the scene would feel (Lane et al., 1990). The advantage of this scale is that it enables an assessment of both conscious and sub-conscious levels of awareness of both one’s own (LEAS-self) and other’s (LEAS-other) emotions (Subic-Wrana et al., 2014). This instrument was shown to be related with a capacity of mentalization, which reflects the ability to interpret ones’ own and other’s feelings, thoughts and intentions (Subic-Wrana et al., 2010).

Participants’ experience of pain intensity and pain sensations was examined by the Brief Pain Inventory (BPI; Radbruch et al., 1999) and the Pain Experience Scale (SES; Geissner, 1995). In order to assess participants’ affective experience and pain during each experimental phase, participants were given a scale for pain and affective experience immediately after each phase. This scale consisted of a visual analog scale for pain, as well as a non-verbal, pictorial affective scale that assesses the pleasure, arousal and dominance aspects of affective experience (Self-Assessment Manikin, SAM; Fischer et al., 2002). SAM was advocated to be a quick and more implicit way of measuring affective experience, particularly because it is a non-verbal cartoon-like graphical assessment of affect (Fischer et al., 2002). It has a nine-point scoring system for measuring pleasure (unhappy to happy), arousal (calm to excited) and dominance (controlled to controlling). Arousal describes the perceived vigilance as a psychological and physical state, while pleasure describes the positive or negative feelings. Dominance describes how much a person feels control in a situation. In addition to SAM, right after the anger task participants were given the state anger subscale of the STAXI, as well as the Experiences in Close Relationship Scale-Revised (ECR-R; Ehrenthal et al., 2009). ECR-R is a validated self-report instrument that assesses attachment anxiety and attachment avoidance in adults (Fraley et al., 2000). For all scales, validated German translations were used.

## RESULTS

### DATA ANALYSIS PROCEDURE

For thermal imaging data, temporal course of the temperature change was included for the statistical analyses. For heart rate and skin conductance levels, the arithmetic mean of the entire data within each experimental phase was computed and then described in detail for each couple.

Firstly, we tested whether experimental condition and participant status (i.e., patient, partner of the patient, and healthy controls) could determine the temporal change of the nose tip and forehead temperature. We applied hierarchical linear models with experimental condition, participant status and temporal course (i.e., number of frames) as fixed effects and the participant as random factor (Singer, 1998). In order to determine the specified characteristics of the temporal course for each participant we added a condition ^*^ temporal course ^*^ participant interaction term to the model. Individual temperature changes were estimated by analyzing each participant separately, and slopes for each condition were computed. For comparisons of slopes between patient and healthy controls, confidence intervals of each slope were computed.

We examined the relationship of emotion regulation and anger regulation with thermal changes by including the scores of the corresponding questionnaires (i.e., LEAS, TAS-20, and STAXI) as covariates in the model. We tested the influence of these psychological measures by introducing them as fixed effects. Additionally, we included a condition ^*^ course ^*^ psychological measure interaction in the model to allow condition specific analyses of their association with the temperature changes. Each psychological domain was tested separately in order to prevent possible effects due to multicollinearity. We did not include the attachment scores in the model due to missing data in Couple 1.

To examine the relationship of physiological processes between partners, based on a previous study (Ebisch et al., 2012), we performed Pearson correlation analyses for nasal tip and forehead temperature between partners for each condition.

In the following sections, firstly, the results of the statistical analyses are presented. Following that, for each couple, the results of heart rate, skin conductance levels, and subjective report measures are described in detail.

### TEMPORAL THERMAL CHANGES ON THE NOSE TIP AND FOREHEAD

**FIGURE 2:**
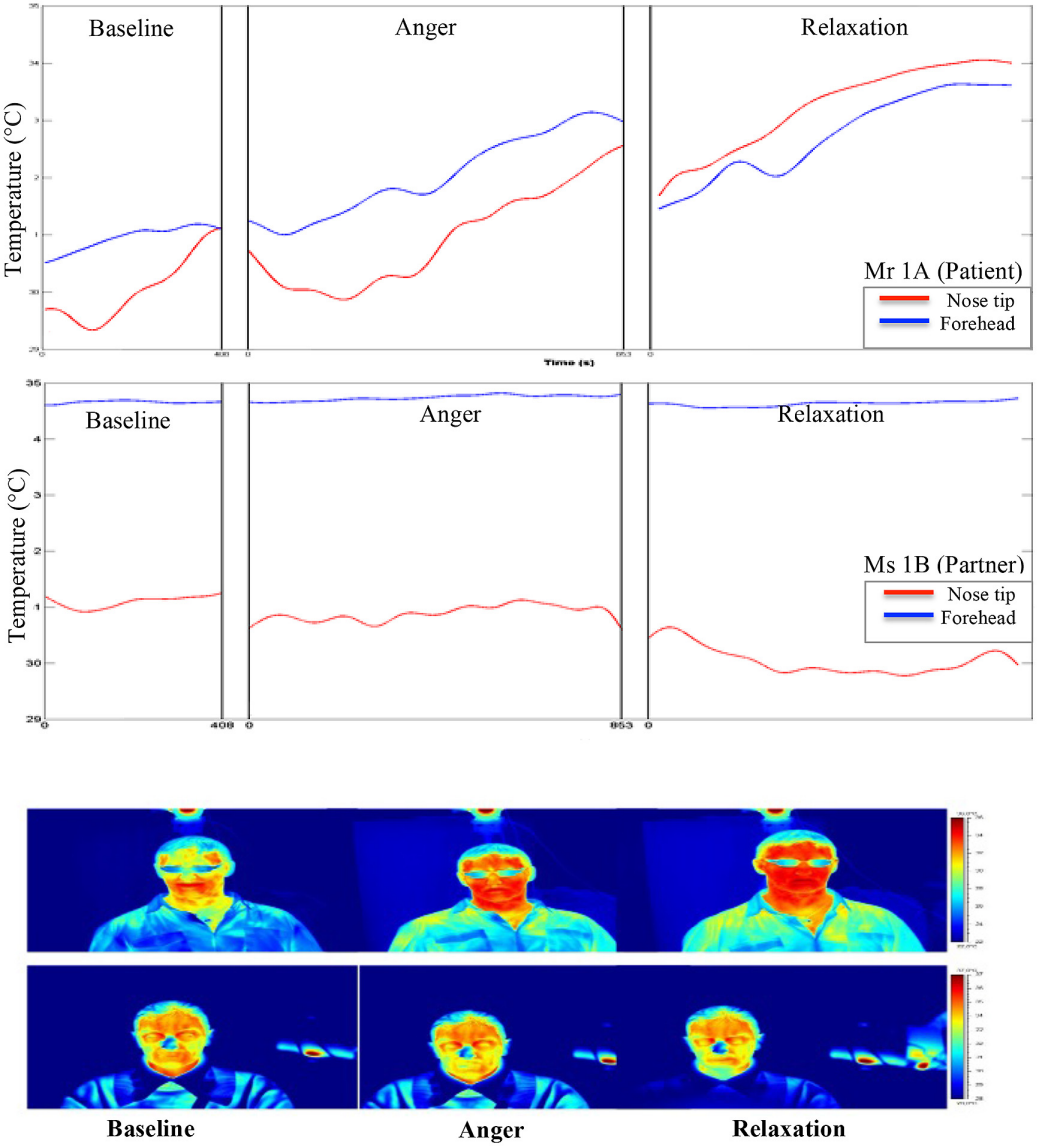
**Graphical and pictorial representations of variations in the facial thermal imprints of Mr 1A and Ms 1B, respectively.** The first illustration belongs to Mr 1A (wears eye glasses) and the second to Ms 1B.

**FIGURE 3:**
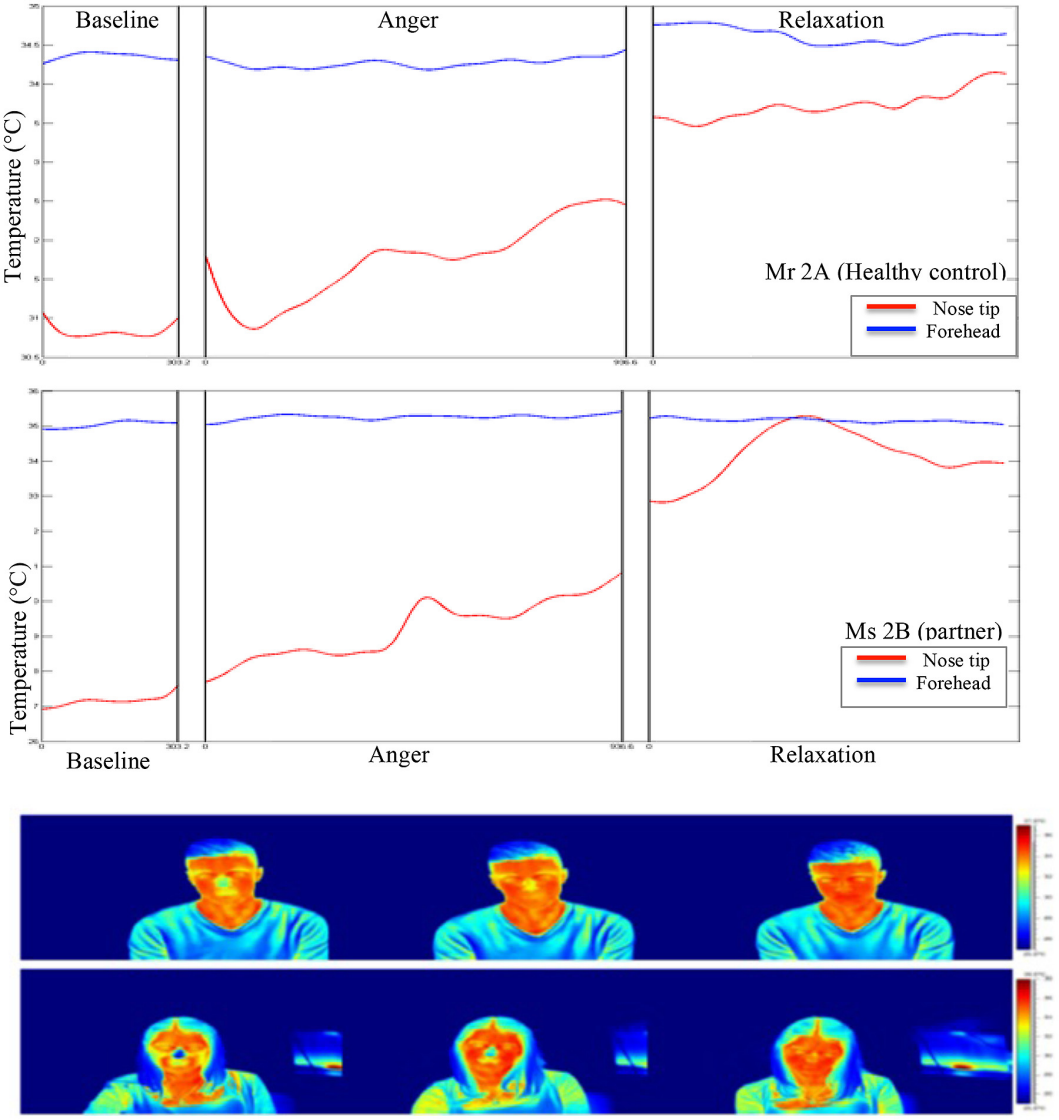
**Graphical and pictorial representations of variations in the facial thermal imprints of Mr 2A and Ms 2B, respectively.** The first illustration belongs to Mr 2A and the second to Ms 2B.

During the experiment, the average skin temperature of the nose tip was rising for all participants except for the patient’s partner, whose nose tip temperature slightly decreased (see **Figures 2** and **3**). The forehead temperature didn’t show a comparable pattern, and related observed changes were comparably small.

All temporal courses were significantly different for each participant for the whole session (see **Table 1**). This could be demonstrated for forehead temperature, too. The full model – again including all participants – confirmed individually different slopes for each condition and all patients. When we compared the slopes of the temperature change between subjects, we found that the forehead temperature of the patient increased significantly in anger and relaxation phases.

**Table 1:** Temporal course of the changes in cutaneous temperature of the participants.

Partner		Baseline	Slope*	Anger	Slope*	Relaxation	Slope*	Sig. p (condition * frame)
		mean (SD)		mean (SD)		mean (SD)		
Patient (Mr 1A)	Nose tip	30.03 (0.55)	0,258	30.93 (0.84)	0.188	33.24 (0.72)	0,182	0.000
	Forehead	30,95 (0.20)	0.099	32,04 (0.71)	0.171**	32,74 (0.73)	0.184**	0.000
Patient’s partner (Ms 1B)	Nose tip	31.09 (0.10)	0.040	30.89 (0.13)	0.023	30.03 (0.24)	-0.035	0.000
	Forehead	34,66 (0.02)	0.002	34,73 (0.05)	0.011	34,62 (0.04)	0.009	0.000
Healthy partner (Mr 2A)	Nose tip	30.82 (0.07)	-0,004	31.74 (0.49)	0,100	33.73 (0.17)	0,042	0.000
	Forehead	34,36 (0.04)	-0.002	34,27 (0.06)	0,008	34,63 (0.10)	-0,016	0.000
Healthy partner (Ms 2B)	Nose tip	27.15 (0.12)	0.069	29.22 (0.81)	0,170	34.18 (0.73)	0,055	0.000
	Forehead	35,04 (0.09)	0.053	35,25 (0.07)	0,007	35,16 (0.05)	-0,011	0.000
Full model								0.000

*Temperature change: degree centigrade per minute. **Temperature changes were significantly higher for the patient, compared to the partners of the healthy couple.

When the psychological factors (i.e., TAS-20, LEAS, STAXI) were included in the model, condition specific associations of these factors with the thermal variations were observed. Although the relationship of the psychological factors with the overall temperature was negligible, high associations were found between these psychological measures and condition specific temperature changes (see **Table 2**). All the psychological factors were significantly associated with temperature changes in each condition, but not with the absolute temperature levels. Changes in the relaxation phase tended to be smaller compared to the initial phases. Higher scores in STAXI and TAS were associated with more pronounced temperature changes. Likewise, lower scores in emotional awareness measured by LEAS were associated with greater temperature changes.

**Table 2:** Psychological predictors of change in nose tip temperature within experimental conditions.

	Overall* b	*p*	b** for temperature change per condition per psychological measure		
			Baseline	Anger	Relaxation
TAS	0,003	0.96	0.0051	0.0027	0.0005
LEAS-self	-0.070	0.42	0.0001	0.0010	0.0015
LEAS-other	-0,043	0.69	-0.0014	-0.0008	-0.0009
LEAS-total	-0.015	0.87	-0.0023	-0.0017	-0.0019
STAXI-trait	0.015	0.85	0,0103	0,0051	0,0001
STAXI-in	0.084	0.93	0,0118	0.0052	-0.0012
STAXI-out	-0,016	0.87	0,0119	0.0056	0.0003
STAXI-control	0,311	0.41	0,0012	-0,0029	-0,0067

*Regression coefficient b; mean temperature predicted by the respective psychological measure. **Regression coefficient b; change in temperature per minute (degree C/min) predicted by the respective psychological measure. In all condition-specific associations with psychological measures: *p* < 0.001.

### CORRELATION OF TEMPERATURE CHANGES BETWEEN PARTNERS

Correlation analysis of nasal tip temperature of the dyads showed significant relationships at *p* < 0.01 (**Table 3**). As forehead temperature did not show much variance across phases, we did not include it in the analysis. At baseline, the nasal tip temperature was positively correlated between the partners of Couple 1 (patient-partner; *r* = 0.89), while for Couple 2 (healthy control-partner) no correlation was found. At anger phase, a positive correlation between the nose tip temperatures of partners of both Couple 1 and 2 was shown (*r* = 0.62 and 0.84, respectively) At relaxation phase a strong negative correlation between the nasal tip temperature of the first dyad (*r* = -0.71) and a weak one (*r* = 0.20) for the second dyad was found.

**Table 3:** Correlation coefficients of the relationship between partners’ nasal tip temperature during each experimental phase.

Couples	Baseline (r_dyads_)	Anger (r_dyads_)	Relaxation (r_dyads_)
Couple 1 (Patient and partner)	0.89^*^	0.62^*^	-0.71^*^
Couple 2 (Healthy control and partner)	-0.007	0.84^*^	0.20^*^

*p < 0.01.

### CASE-BASED ANALYSES

#### Couple 1: patient and partner

***Pain and psychological symptoms.*** Mr 1A (patient) suffered from somatoform pain disorder. His pain encompassed a chronic widespread pain and local pain, which is elicited by stimuli that normally don’t provoke pain (i.e., allodynia). The pain concentrated especially on his back, arms, legs, and joints that has strongly impaired his life for more than 5 years. In the last 2 weeks, he had very intense level of pain that had affected his overall activity, his work, as well as his relationships with others. His level of affective pain, meaning his evaluative and emotional reaction to pain, was very high and fell within the 100th percentile of the normative sample of pain patients. On the other hand, his level of sensory pain, that is, his perceptual ratings of pain intensity fell within the 46, 2% of the normative pain patient sample. The patient described a moderate level of depressive state characterized by sadness, hopelessness, and little interest or joy in life. He took the medications of duloxetine (a serotonin-norepinephrine reuptake inhibitor), amitriptyline (a tricyclic antidepressant) and quetiapine (a short acting atypical antipsychotic).

Ms 1B did not report experiencing pain except a little pain in some body parts that affect her at a minimum level. She described her health as very good although she reported some general life stress to a little extent and some relationship difficulties with her partner.

***Emotion regulation reports.*** The TAS-20 reports of Mr 1A classified him as alexithymic (raw score = 65) according to the cut-off scoring method, which indicated his difficulties in identifying and describing his feelings. Supporting this finding, his total level emotional awareness score (LEAS_sumscores_ = 47, *M* = 2.35) measured by LEAS put him around the 12th percentile of the healthy men sample (Subic-Wrana et al., 2001). This mean LEAS-total score corresponded to the range of scores of somatoform patients in a previous study (*M* = 1.93, SD = 0.58; Subic-Wrana et al., 2010). Moreover, according to a recent evaluation criterion of four item LEAS (Subic-Wrana et al., 2014), his mean score of LEAS not_but-total denoted his emotional awareness at an implicit level (i.e., a preconscious level of emotional awareness, that the affective arousal is expressed as bodily sensations or action tendency). Similarly, his mean scores for awareness of his own emotions (LEAS-self) and for other (LEAS-other) were 2.2, which again indicated an implicit level of emotional awareness (see **Table 4**, for subjective reports of the participants).

**Table 4:** Participants’ scores for measures of emotion regulation and attachment styles.

Subject	TAS-20 total	LEAS total (mean)	LEAS-self (mean)	LEAS-other (mean)	STAXI-trait anger	STAXI anger-out	STAXI anger-in	STAXI anger-control	ECR-R anxiety	ECR-R avoidance
Mr 1A	65	47 (2.35)	44 (2.2)	44 (2.2)	30	29	29	25	–	–
Ms 1B	37	59 (2.95)	57 (2.85)	45 (2.25)	20	19	12	24	–	–
Mr 2A	44	61 (3.05)	49 (2.45)	53 (2.65)	22	16	12	24	1,72	1,72
Ms 2B	38	66 (3.3)	62 (3.1)	60 (3)	18	11	11	21	3,44	1,55

*TAS-20, Toronto Alexithymia Scale-20; LEAS, Level of Emotional Awareness Scale; STAXI, Spielberger Anger Expression Inventory; Experiences in Close Relationships-Revised (ECR-R).*

In terms of anger regulation, he reported high trait-anger, which means a general disposition to become angry (within the 99th percentile of the men sample). He reported expressing anger in a poorly controlled manner (99th percentile) or suppressing his anger (99th percentile). Yet, his expenditure of energy to monitor and control his anger was at a moderate to high level (70th percentile).

Ms 1B’s TAS-20-based alexithymia score (raw score = 37) indicated her good ability to be aware of her feelings, and to identify and describe them. Similarly, her total LEAS score (LEAS _sum_ _scores__=_ 0 59, *M* = 2.59) put her into the 35th percentile of women sample and almost on a level of explicit emotional awareness, indicating her ability to experience emotions consciously and express them verbally (Subic-Wrana et al., 2014). Interestingly, her mean LEAS-self (*M* = 2.85) and LEAS-other (2.25) scores were quite discrepant from each other compared to other participants of our study. Her LEAS-other score was almost at an implicit level of emotional awareness.

Her anger scales showed a moderate to high level of trait anger (75th percentile of the women sample). She reported a high tendency to suppress anger expression (80th percentile) and low-moderate tendency (50th percentile) to express anger in an outwardly negative and poorly controlled manner. She also reported a moderate to high (70th percentile) level of effort to monitor and regulate her anger.

***Heart rate (HR) and skin conductance levels (SCL).*** The mean HR of Mr 1A, which was greater compared to his partner, was did not change much from baseline (*M* = 101.8, SD = 4.7, Min = 83.5, Max = 112.1) to anger (*M* = 101, SD = 5, Min = 83.5, Max = 110.5) but decreased at relaxation phase (*M* = 90.3, SD = 2.7, Min = 81.7, Max = 97.8) while the HR of Ms 1B remained relatively stable at almost all phases (Baseline: *M* = 71.3, SD = 5.4, Min = 56, Max = 86.2; Anger: *M* = 72.9, SD = 6.3, Min = 53.3, Max = 91.9; Relaxation: *M* = 69, SD = 6, Min = 56.9, Max = 101).

**FIGURE 4:**
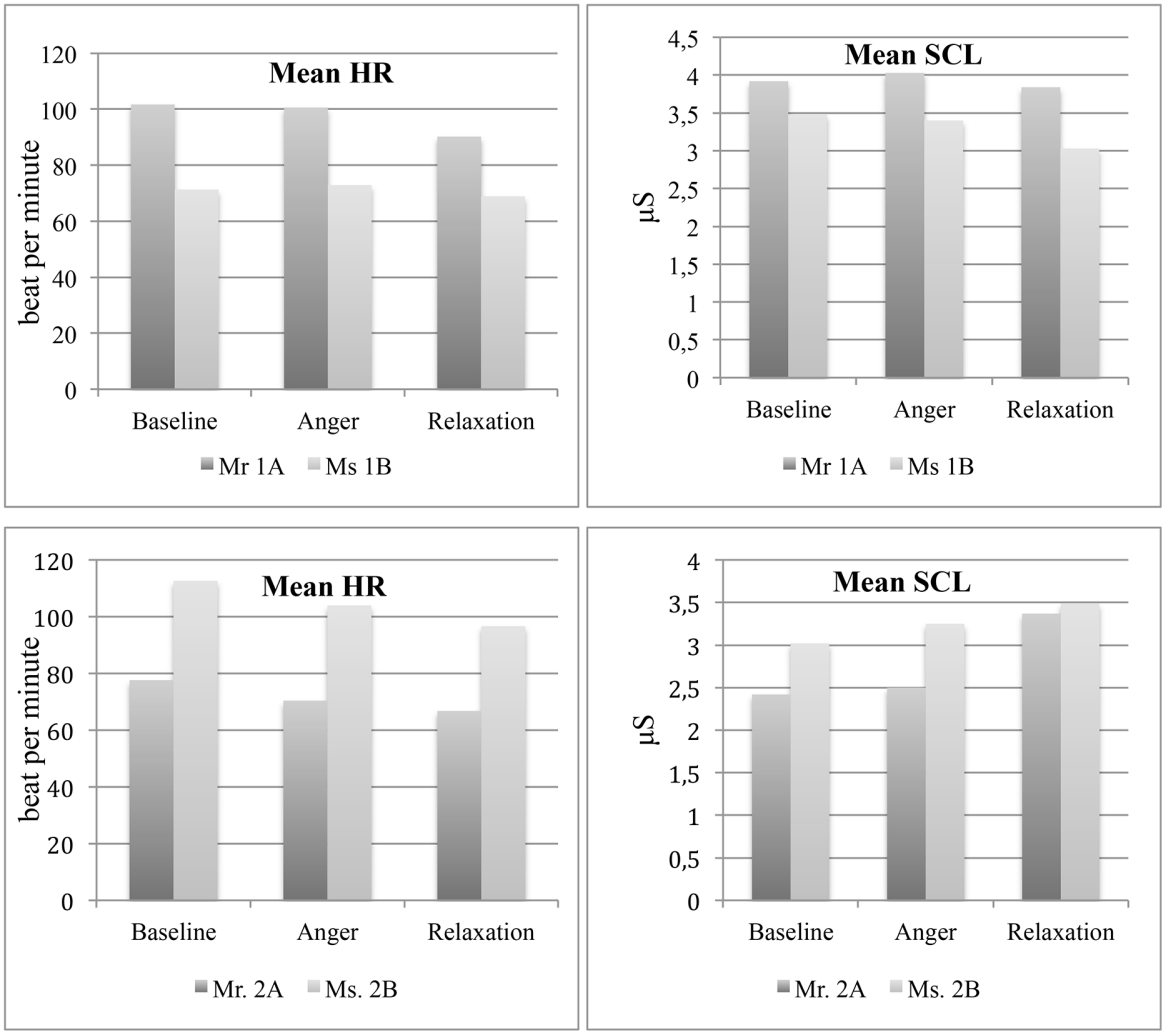
**Mean heart rate and skin conductance levels of the couples at three experimental phases**.

With regard to SCL, Mr 1A showed a slight increase from baseline (*M* = 3.9, SD = 0.1, Min = 3.7, Max = 4.7) to anger phase (*M* = 4.0, SD = 0.2, Min = 3.7, Max = 4.9) and then a decrease at relaxation phase (*M* = 3.8, SD = 0.2, Min = 3.4, Max = 6.5). On the other hand, Ms 1B showed a slight decrease from baseline (*M* = 3.5, SD = 0.2, Min = 3.05, Max = 4.04) to anger phase (*M* = 3.4, SD = 0.2, Min = 3.1, Max = 4.1), and a much pronounced decrease at relaxation phase (*M* = 3.03, SD = 0.5, Min = 2.68, Max = 5.69; see **Figure 4**).

#### State-affective experience

Mr 1A reported a pronounced increase in pain experience at relaxation phase compared to other phases. In terms of experience of pleasure, arousal and dominance, he reported himself quite unhappy, a bit aroused and a bit being controlled at almost all phases, which did not show much variance (see **Table 5**, for the affective experience ratings for self and other). He evaluated his partner’s affect similar to his own, as quite unhappy and a bit aroused. In terms of dominance, he reported Ms 1B as quite controlling at baseline, while his interpretation of her dominance decreased at anger and relaxation phase.

**Table 5:** Affective experience ratings of the participants for self and other.

	Affect								
	Pleasure			Arousal			Dominance		
	Base	Anger	Relax	Base	Anger	Relax	Base	Anger	Relax
Mr 1A (self*)	3	4	4	4	4	4	4	4	4
Mr 1A (other*)	3	4	4	3	3	4	6	4	4
Ms 1B (self)	9	3	8	1	6	1	1	4	2
Ms 1B (other)	8	4	7	2	5	2	1	8	4
Mr 2A (self)	9	5	8	3	1	1	7	6	8
Mr 2A (other)	9	3	8	5	6	1	7	5	8
Ms 2B (self)	8	7	8	4	5	2	5	3	5
Ms 2B (other)	8	7	7	4	4	2	5	4	5

*“Self” stands for participant’s report about own affective experience. “Other” stands for participant’s evaluation of partner’s affective experience.

Ms 1B reported more differing affective experience for herself and her partner among phases. At baseline, she reported to be happy and her arousal and dominance were at low levels, while she reported feeling quite unhappy, a bit aroused and a bit controlled at anger phase. At relaxation phase, she reported feeling happy, relaxed and a bit controlled. Similar to her own ratings, she rated her partner also as quite happy, relaxed and controlled at baseline. At anger phase she appraised Mr 1A’s affective experience also similar to her own, quite unhappy, a bit aroused but quite controlling. At relaxation phase, she also rated her partner as relaxed again with higher level pleasure and an average level of dominance.

#### Case 2: healthy control and partner

***Pain and psychological symptoms.*** The couple presented no pronounced complaints about pain, depression, anxiety or stress. In addition, they described their health condition as very good. They did not report any chronic disease or use of medication.

***Emotion regulation reports.*** According to the TAS scores, both partners reported a good ability in identifying and describing feelings and were in the range of no-alexithymia according to the cut-off scoring. Yet, Mr 2A had a slightly higher score than his partner (TAS 20 total raw scores were 44 and 38, respectively for Mr 2A and Ms 2B; see **Table 4**). Parallel with the TAS scores, the total level of emotional awareness of Mr 2A was around 45th percentile of the healthy man sample (LEAS-total raw score = 61). Yet, Mr 2A was the only participant who had lower LEAS-self score (*M* = 2.45) than the LEAS-other (*M* = 2.65) score, which indicated almost an implicit level of emotional awareness. The LEAS-total score of Ms 2B was also consistent with her TAS score and she could be placed within the 52th percentile of healthy women sample. In addition her LEAS-self and -other scores counted her at an explicit level of emotional awareness (*M* = 3.1 and 3, respectively).

Regarding anger regulation, Mr 2A reported a high level of trait anger (80th percentile). His tendency to suppress anger, to express anger negatively and to try to control and modulate his anger was at moderate level (55, 55, and 60th percentile, respectively). On the other hand, Ms 2B had a moderate level of trait anger (55th percentile) and anger modulation (50th percentile), but a low level of suppressing anger (15th percentile) and expressing anger negatively (40th percentile).

According to the ECR-R, which assesses attachment styles, Ms 2B had a high score in anxious attachment style (*M* = 3.44), which was within the range of clinical sample in a previous validation study with German sample (*M* = 3.08, SD = 1.27, Ehrenthal et al., 2009). The ECR-R scores of Mr 2B were within range of healthy controls.

***Heart rate and skin conductance levels.*** The mean HR of both partners decreased from baseline (Mr 2A: *M* = 77.6, SD = 6.3, Min = 60, Max = 101; Ms 2B: *M* = 112.6, SD = 5.2, Min = 96, Max = 120) to anger (Mr 2A: *M* = 70.4, SD = 6.5, Min = 56, Max = 89; Ms 2B: *M* = 100.4, SD = 6.5, Min = 76, Max = 112), and then to relaxation phases (Mr 2A: *M* = 66.8, SD = 5.6, Min = 52, Max = 88.8; Ms 2B: *M* = 97.2, SD = 5.4, Min = 76.8, Max = 109.7). On the other hand, mean SCL increased in both partners from baseline (Mr 2A: *M* = 2.4, SD = 0.03, Min = 2.37, Max = 2.53; Ms 2B: *M* = 3.02, SD = 0.02, Min = 2.86, Max = 3.76) to anger (Mr 2A: *M* = 2.50, SD = 0.04, Min = 2.38, Max = 2.59; Ms 2B: *M* = 3.25, SD = 0.17, Min = 3.01, Max = 3.7), with a more pronounced increase at relaxation phase (Mr 2A: *M* = 3.37, SD = 0.11, Min = 3.28, Max = 3.68; Ms 2B: *M* = 3.49, SD = 0.07, Min = 3.04, Max = 3.72; see **Figure 4**).

***State-affective experience.*** Mr 2A reported his pleasure level to decrease at anger phase. Yet, his arousal and dominance levels did not vary much across phases, depicting almost a relaxed and dominant state. However, he appraised his partner’s pleasure and arousal quite changing and compatible with the experimental phases. He reported his partner’s pleasure level as decreasing and arousal as increasing at anger phase and then vice versa at relaxation phase. Like in Couple 1, anger task was the only phase when he felt himself more dominant compared to his partner.

Ms 2B reported very few variances in terms of her own, and her partner’s pleasure levels, remaining almost stable across phases. She reported herself and her partner feeling quite happy. On the other hand, she reported both herself and her partner a bit aroused at baseline and anger phases and then relaxed at relaxation phase. Consistent with her partner’s appraisal, she felt being less dominant compared to her partner at anger phase.

## DISCUSSION

The theoretical accounts of SSD accentuate a network of bi-directional relationships between interpersonal interactions, emotion regulation and bodily disturbances (Waller and Scheidt, 2006; Henningsen et al., 2007; Subic-Wrana et al., 2010; Luyten et al., 2012). Despite this close linkage, there are only a few available studies having examined the real-time, affective interpersonal interactions of patients with SSD (e.g., Merten and Brunnhuber, 2004; Cano et al., 2008; Leong et al., 2011). These studies have shown that, both partners in an ongoing interaction reciprocally contribute to emotion regulation process, which becomes a precipitating and maintaining factor for the somatic symptoms. However, the literature is scarce of empirical research that have examined the coordination of multiple components of emotion (i.e., physiology, behavior, experience) of both parties in a real-time dyadic interaction.

In this case study, we aimed to examine how intra- and interpersonal emotion regulation at physiological and experiential levels is related to SSD. Previous studies suggest some kind of discordance between physiological, experiential and behavioral components of emotional process in SSD (Ditzen et al., 2008; Luyten et al., 2011; Pollatos et al., 2011b; Bondo-Lind et al., 2014; Okur, et al., in revision). In line with earlier studies, we proposed that the patient would present an intrapersonal incoherence among emotion response systems, characterized by higher autonomic activity but restricted affective experience compared to healthy controls. Moreover, trait emotion regulation patterns would affect the physiological changes during the affective interactions. At the interpersonal level, we predicted that, emotional incoherence would be more likely to be reciprocated by a complementary incoherence of emotional processing in the partner. This pattern would generate an interpersonal emotional incoherence represented by low correlations between partners in terms of physiological and experiential emotional processing.

In this paper, following an introduction of the accounts of emotion regulation in SSD, we presented an interpersonal experimental paradigm that included two case couples consisting of a patient with somatoform pain and his partner, and a couple of healthy controls. We chose anger and positive affect as central affects since these were reported to play particular roles in chronic pain (Fernandez and Turk, 1995; Zautra et al., 2005; van Middendorp et al., 2010). We measured participants’ cutaneous temperature, heart rate, and skin conductance levels as imprints of autonomic activity during the interaction. Besides, we examined self-report and performance-based emotion regulation, affective experience and attachment styles of the participants. We investigated not only participants’ own affect but also their perception of their partner’s affective experiences.

The paradigm was successful in generating physiological and experiential changes in an ecologically valid and a structured interpersonal setting, which allowed for a dynamic emotional interaction. Trait emotion regulation, namely, alexithymia, level of emotional awareness and anger regulation predicted the course of cutaneous temperature changes across phases. The patient, his partner and the healthy couple showed some distinctive patterns of emotion regulation, as well. However, it should be noted that the results should be interpreted cautiously as we examined only two cases in this study.

The temporal analysis of the course of temperature changes on nose tip and forehead showed significant variances across phases, pointing to the effectiveness of experimental manipulation. Nasal tip temperature increased from baseline to relaxation in all participants except the patients’ partner, whose nasal tip temperature slightly decreased. This regulation pattern of the patients’ partner might suggest a complementary down-regulation of physiology in her interaction with the patient, who showed higher autonomic activity. In fact, as predicted, the patient showed higher stress responses as compared to his partner and healthy controls depicted by significant temperature increase on forehead in anger and relaxation phases. In addition, his mean SCL and HR were higher than his partner throughout the experimental phases. Such vigilant autonomic activity in SSD has been shown in previous studies, as well (Seignourel et al., 2007; Burns et al., 2008; Twiss et al., 2009; Luyten et al., 2011; Pollatos et al., 2011a,b).

Trait emotion regulation patterns also predicted the course of temperature changes. Higher alexithymia, increased anger regulation difficulties and lower scores in emotional awareness predicted higher changes in nasal tip temperature. This result supports the previous findings that have connected emotion regulation deficits with aberrant and higher physiological stress responses (Luyten et al., 2011; Pollatos et al., 2011a,b). Parallel with this finding, as expected, the patient had more restricted awareness and reflection to his own and others’ emotions as well as high trait anger and poor anger regulation. The partner of the patient also showed a moderate to high level of trait anger. This prevailing angry feeling in both partners may reflect the contagious nature of affects in interpersonal interactions (Hatfield et al., 1993). The patient’s affective pain, which indicates the evaluative and emotional reaction to pain, was also at a very high range although his sensory pain was at a moderate level. This illustrates that somatoform patients’ affective appraisals regarding symptoms may contribute to the amplification of the symptoms (Hadjistavropoulos and Craig, 1994).

The second couple consisting of healthy partners showed indications of relatively enhanced emotion regulation. They both showed greater ability of being aware of, identifying and describing their own and other’s emotions. However, some degree of trait anger existed in both partners’ reports classifying Mr 2A as having high trait anger and a moderate level of anger regulation difficulties and Ms 2B as having a moderate level of trait anger.

The relationship of state affective experience and accompanying physiological changes were quite distinctive between participants. Although the patient’s cutaneous temperature, HR and SCL showed noticeable variations across experimental phases, he reported quite stable and moderate level of arousal and pleasure, which were inconsistent with his higher autonomic reactivity. This discrepancy points to incoherence between his affective experience and somatic concomitants. In fact, the patient’s high alexithymia and low emotional awareness scores could explain his restricted access to his feelings and accompanying autonomic changes. The subjective reports of Ms 1B, on the other hand, were, as expected, much more consistent with her physiological changes except for the baseline. The lack of consistency at baseline might be due to the possible performance stress at the beginning as well as her attempt to give a desired response suitable to a neutral baseline task.

For the partners of the control couple, the concordance of the subjective reports and physiological changes seemed to be superior than the patient to a certain extent. At anger phase, the nasal tip temperature and mean SCL increased in both partners, and they both reported a decrease in pleasure. Ms 2B reported that her arousal rose at anger phase, which was accompanied by a rise in nasal tip temperature and mean SCL although her mean HR declined. At relaxation phase, she reported lower arousal but her values of physiological imprints except her decreasing mean HR continued to increase. However, Mr 2A reported few changes in terms of arousal and pleasure, despite his declining mean HR and increasing mean SCL and thermal imprints from baseline to relaxation. Explaining this discordance, he scored low in LEAS-self subscale indicating some difficulties in consciously experiencing and describing his own emotions.

Analyses of interpersonal level of emotion regulation brought forward more multifaceted results than we proposed. The graphical trends of temporal changes in nasal tip temperature suggested discordance between the patient-partner dyads (Couple 1) and concordance in healthy control-partner dyads (Couple 2). However, correlation analysis of these temporal courses between partners, which are apparently more sensitive to the changes than visual inspection, suggested more concordance between the first dyad compared to the second one. At baseline, a positive correlation between nose tip temperatures of the partners was found only in the first couple. At anger phase, the partners of both Couple 1 and 2 presented strong positive correlations in nose tip temperature. At relaxation, only between the first couple, a strong negative correlation of temperature change was found. These findings might suggest a pattern of interpersonal emotion regulation in patients with SSD, which is quite the reverse of our predictions. The patient and his partner seem to show more interrelated change of temperature compared to the control couple.

The strong correlations of temperature between the first couple might be explained with the reciprocal nature of social interactions, which connotes the adaptive and complementary behavior of the interaction partner. It might be speculated that, by down-regulating the physiological responses, the partner of the patient complemented the patient’s higher autonomic activity and vice versa. In fact, the couple’s affective reports for self and other lend some support to this complementarity. While the patient reported experiencing almost similar levels of pleasure and arousal, his partner reported experiencing more variance in these domains. Moreover, the patient underrated his partner’s pleasure and arousal levels, while his partner overrated these affective experiences of him. The couple’s poor performance on recognizing the other’s affective experience was consistent with previous studies, which have reported emotion recognition difficulties in patients with SSD (Pedrosa Gil et al., 2008; Beck et al., 2013). Supporting these findings, both the patient and his partner had low scores in LEAS-other, which implies difficulties in understanding the other’s emotions at an explicit level (Subic-Wrana et al., 2014).

The second couple with the healthy partners performed well in LEAS-other subscale, which implies a better ability of consciously recognizing the other’s emotions compared to the first couple. They also performed relatively better in perceiving the trend of affective change in the partner. They correctly appraised each other’s arousal to decrease at relaxation phase, and dominance to lessen at anger and rise at relaxation phases. Mr 2A was also accurate in perceiving the rise of his partner’s arousal at anger although Ms 2B was not. The couple also could not accurately evaluate the changes in the other’s feeling of pleasure. It seems that Mr 2A attributed some emotionality and fluctuating emotional responses to his partner. It may be speculated that the anxious attachment style of Ms 2B could contribute to her partner’s attributions.

Our study has a number of limitations. Although our study demonstrates how embodied and intersubjective emotion models can be integrated into psychosomatic research, it involves only two cases and therefore provides scarce evidence for our hypotheses. Future research with greater sample size and robust statistical methods should examine the affective processes of interacting couples empirically. Secondly, despite previous recommendations (Mauss et al., 2005), in order not to interrupt the interactions of the couples, we could not include continuous measures of subjective experience. Thirdly, since we included only two case couples, we did not statistically analyze the continuous temporal changes of SCL and electrocardiography at within and between partners. Nevertheless, we demonstrated a tentative example to examine the relationship between emotion regulation and temporal course of cutaneous temperature changes at intra- and interpersonal levels. Forthcoming research should adopt statistical approaches with high temporal sensitivity (e.g., time sequence analysis, cross correlation analysis, actor independence models) in order to examine the course and coordination of multiple emotion response systems at these multi-levels (Hollenstein and Lanteigne, 2014). Likewise, we did not use observational measures of emotional interaction that allows for temporal analyses between observational and physiological data. We plan to employ observational measures for assessing emotion regulation and affective interactions in our ensuing study. Finally, future research should statistically control for sex differences and use of medication as they can have potential effects on emotional processing and physiology. Also, because factors, such as pain and alexithymia can be confounded with the patient status, ceiling or bottom effects are possible. Therefore causal assumptions should be made tentatively.

Our study illustrates the scientific yield of an embodied interpersonal paradigm for studying emotion regulation in SSD, in particular for regulation of anger and positive affect. An enhanced understanding of this intra- and interpersonally, and dynamically regulated phenomenon will provide potential for an optimized clinical regime and psychotherapy.

## Conflict of Interest Statement

The authors declare that the research was conducted in the absence of any commercial or financial relationships that could be construed as a potential conflict of interest.
